# Ketone Diester Ingestion Impairs Time-Trial Performance in Professional Cyclists

**DOI:** 10.3389/fphys.2017.00806

**Published:** 2017-10-23

**Authors:** Jill J. Leckey, Megan L. Ross, Marc Quod, John A. Hawley, Louise M. Burke

**Affiliations:** ^1^Centre for Exercise and Nutrition, Mary MacKillop Institute for Health Research, Australian Catholic University, Melbourne, VIC, Australia; ^2^Sports Nutrition, Australian Institute of Sport, Canberra, ACT, Australia; ^3^ORICA-BikeExchange (WorldTour Team), UCI, Adelaide, SA, Australia; ^4^Research Institute for Sport and Exercise Sciences, Liverpool John Moores University, Liverpool, United Kingdom

**Keywords:** ketone ester, cycling, time-trial, performance, ketones

## Abstract

We investigated the effect of pre- “race” ingestion of a 1,3-butanediol acetoacetate diester on blood ketone concentration, substrate metabolism and performance of a cycling time trial (TT) in professional cyclists. In a randomized cross-over design, 10 elite male cyclists completed a ~31 km laboratory-based TT on a cycling ergometer programmed to simulate the 2017 World Road Cycling Championships course. Cyclists consumed a standardized meal [2 g/kg body mass (BM) carbohydrate (CHO)] the evening prior to a trial day and a CHO breakfast (2 g/kg BM CHO) with 200 mg caffeine on the morning of a trial day. Cyclists were randomized to consume either the ketone diester (2 × 250 mg/kg) or a placebo drink, followed immediately by 200 mL diet cola, given ~ 30 min before and immediately prior to commencing a 20 min incremental warm-up. Blood samples were collected prior to and during the warm-up, pre- and post- TT and at regular intervals after the TT. Urine samples were collected pre- and post- warm-up, immediately post TT and 60 min post TT. Pre-exercise ingestion of the diester resulted in a 2 ± 1% impairment in TT performance that was associated with gut discomfort and higher perception of effort. Serum β-hydroxybutyrate, serum acetoacetate, and urine ketone concentrations increased from rest following ketone ingestion and were higher than placebo throughout the trial. Ketone ingestion induces hyperketonemia in elite professional cyclists when in a carbohydrate fed state, and impairs performance of a cycling TT lasting ~50 min.

## Introduction

Substrate utilization during exercise is influenced by several factors including the relative intensity and duration of exercise, an individual's training status and the effect of the preceding diet on both the substrate pool and the prevailing hormonal milieu (Hawley et al., 2015). As exercise intensity increases, there is a greater reliance on carbohydrate (CHO) based fuels (i.e., muscle and liver glycogen, blood glucose, lactate) and a reduction in the utilization of fat substrates (Brooks and Mercier, 1994). Ketone bodies provide another potential source of readily oxidized fuel for skeletal muscle, but are predominately associated with conditions of metabolic stress such as starvation, where they are needed to preserve essential function of peripheral tissues including the brain and heart (Robinson and Williamson, 1980; Veech, 2004). However, there has been recent interest in the notion that increasing blood ketone concentrations could contribute to an enhancement of exercise performance by providing a readily available, alternative oxidative substrate for working muscle, sparing the limited stores of muscle glycogen (Pinckaers et al., 2017). As such, models of hyperketonemia from endogenous origin such as chronic exposure to a ketogenic diet (Burke et al., 2017) as well as introduction of exogenous sources of ketone bodies from ketone ester supplements (Cox et al., 2016) have been investigated. Nevertheless, there is some concern that the presence of high circulating concentrations of ketone bodies could inhibit the flux of other muscle substrates, either by impairing (rather than “sparing”) muscle CHO oxidation and/or inhibiting adipose tissue lipolysis (Evans et al., 2017). As such, the situations in which an available ketone supply may benefit exercise capacity or performance may be determined by the duration and/or intensity of exercise and the need for combinations of muscle substrate to meet the metabolic demands.

Ketone bodies, namely D-β-hydroxybutyrate (βHB), acetone and acetoacetate (AcAc), are produced in the liver mitochondria from acetyl-CoA in response to an increased mobilization of free fatty acids (FFA) from adipose tissue in situations of reduced CHO availability (Robinson and Williamson, 1980). As summarized in recent reviews (Egan and D'Agostino, 2016; Pinckaers et al., 2017), under conditions of high CHO availability, circulating concentrations of ketone bodies are low, but are slightly elevated (0.1–0.5 mmol/L) by an overnight fast and further raised by exercising in a fasted state (0.5–1.0 mmol/L). Prolonged fasting/starvation (5 days) causes a maximal increase in rates of ketone body production (1–2 mmol/min or 140–280 g/day), leading to increased plasma concentrations that plateau under normal physiological conditions at ~7–10 mmol/L. Meanwhile, chronic exposure to a ketogenic diet [low CHO (<50 g/d), low-moderate protein (~15% of energy), high fat (75–80% of energy)] raises plasma ketone bodies to 1–2 mmol/L after several days, with concentrations reaching the apparent plateau achieved by prolonged fasting, according to the level of CHO restriction and duration of “keto-adaptation” (Pinckaers et al., 2017). Exogenous forms of ketone bodies include ketone salts, and more recently, ketone esters. Ingestion of the former appears to be less effective in increasing circulating ketone body concentrations and carries a significant salt load (Balasse and Ooms, 1968). Recently, a newly produced ketone monoester, R-3-hydroxybutyl R-3-hydroxybutyrate (Clarke et al., 2012), increases in plasma ketone concentrations (3–6 mmol/L) within the hours following its ingestion (400–600 mg/kg BM), although this may be altered by concomitant intake of food (Evans et al., 2017; Pinckaers et al., 2017).

To investigate the potential benefits to metabolism and sports performance, Cox et al. (2016) studied the effects of ingesting either CHO or CHO plus ketone ester (573 mg·kg^−1^ BM) on performance in trained cyclists. Their ingestion protocol induced a higher blood D-βHB concentration during submaximal cycling (ranging between ~1.5 and 3 mmol/L) and lead to a subsequent improvement in time-trial (TT) performance by ~2% following ketone ester and CHO ingestion compared to the ingestion of only CHO. However, aspects of the study design are inconsistent with conditions of “real world” cycling competition. Accordingly, we examined the effect of pre-“race” ingestion of a ketone diester on blood ketone body concentrations, substrate metabolism and performance under conditions of elite professional cycling; ingestion of a pre-race CHO-rich meal, inclusion of a warm-up, involvement of world-class cyclists and simulation of a real-life course. We hypothesized that this protocol would result in acute nutritional ketosis but that no performance improvement would be observed due to the high intensity nature of a real-life TT event which is dependent on the high rates of energy production from the oxidation of CHO-based fuels.

## Materials and methods

### Ethical approval

This study conformed to the standards set by the *Declaration of Helsinki* and was approved (#20161005) by the Ethics Committee of the Australian Institute of Sport (AIS). After comprehensive details of the study protocol were explained to the participants verbally and in writing, all participants provided written informed consent.

### Overview of study design

**Figure 1:**
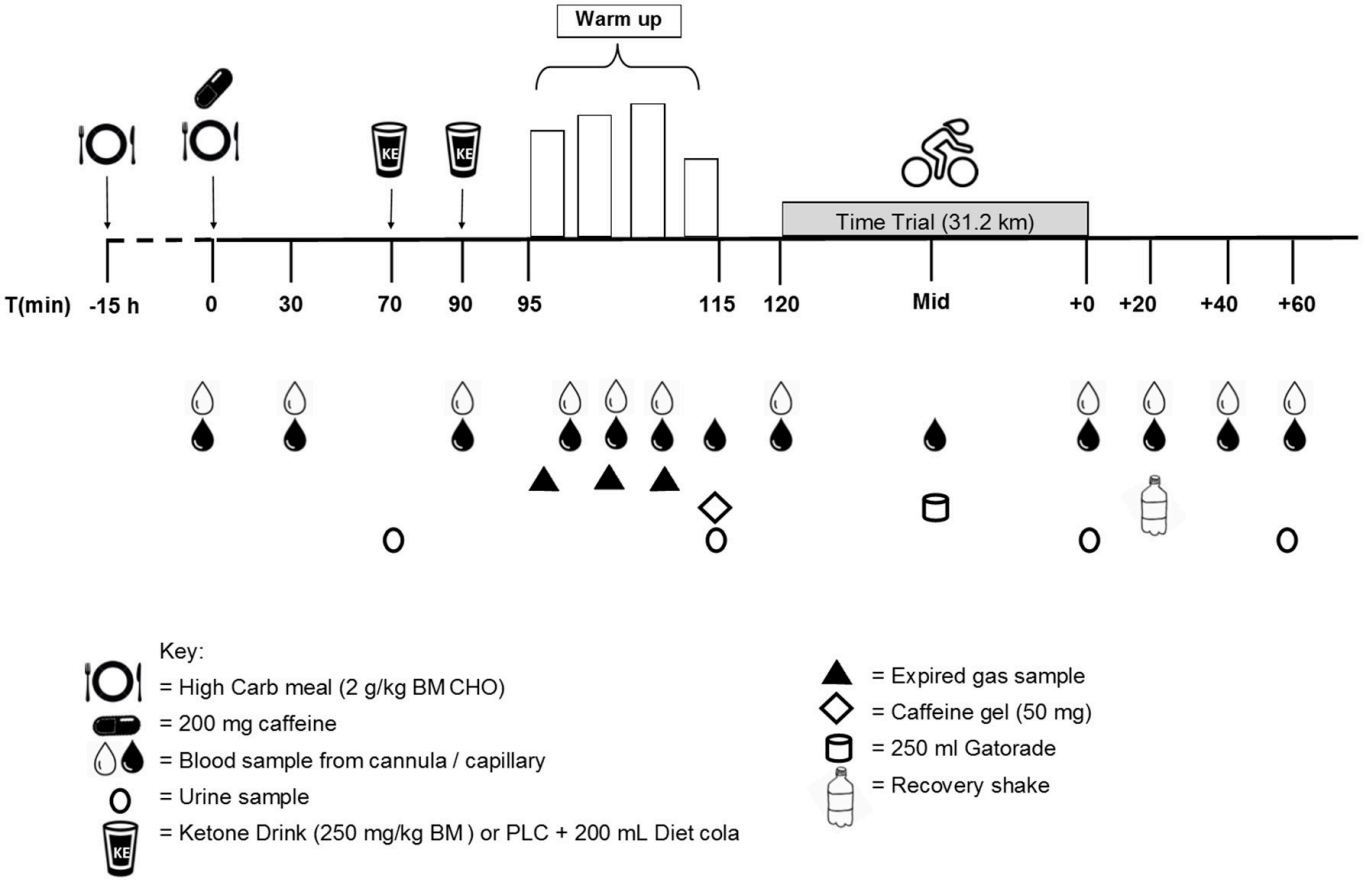
Overview of study design.

The study was a randomized crossover, double-blind, placebo-controlled design using elite (professional) cyclists attending a pre-season camp at the AIS, Canberra. On two separate occasions, three days apart, participants completed a 20-min standardized warm-up and rested for 5 min prior to completing a 31 km TT performed on a cycling ergometer (Figure 1). Participants were randomized to consume a 1,3-butanediol acetoacetate diester (described subsequently; KET; two doses of 250 mg/kg BM) or a viscosity and color-matched (PLAC) drink, given ~30 min before and immediately prior to commencing the warm up. It was not possible to completely replicate the taste of the KET drink, but a comparably novel and bitter-tasting PLAC was prepared from a mixture of flavor essences (rum, almond, and bitters Angostura). Pilot testing revealed that the intake of a small volume of diet cola immediately after the KET and PLAC was able to quickly mask the taste and texture of the previous drink. In any case, none of the participants had previously ingested a ketone ester supplement and were therefore unable to recognize its characteristics. In recognition of the World Anti-Doping Code under which these cyclists compete, it was ascertained that ketone supplements are not considered a prohibited substance by the World Anti-Doping Agency.

### Participants

Eleven internationally competitive male cyclists [age, 25 ± 7 (*SD*) y; body mass (BM), 73.7 ± 7.6 kg; V°O_2_peak, 71.4 ± 5.6 mL/kg/min, 5.3 ± 0.3 L/min; Maximal Aerobic Power (MAP), 494 ± 20 W] from the ORICA-BikeExchange UCI World Tour (Road Cycling) team participated in this study. Participants included world class elite (*n* = 8; e.g., 2016 Paris-Roubaix winner, stage medalists from Tour de France, Tour Down Under, Giro d'Italia, Vuelta a España and Australian National championship Time Trial medalists) and highly trained under 23 riders contracted to the team (*n* = 3).

### Preliminary testing and familiarization

Before commencing the experimental phase, participants visited the laboratory to complete an incremental exercise test and a familiarization with the cycling ergometer (Velotron, Racermate Inc., Seattle, WA, USA) and the experimental exercise protocol (simulated 2017 World Championships time trial course, Bergen Norway).

### Incremental cycling test

Participants completed a 5-min warm-up at 150 W on the cycle ergometer (Lode Excalibur Sport, Groningen, The Netherlands), thereafter the test protocol started at 180 W and increased 30 W every 60 s until volitional exhaustion. MAP was determined as the power output (PO) of the highest stage completed. If the participant finished partway through a 60 s stage, MAP was calculated in a pro-rata manner. During the maximal test, expired gases were collected into a calibrated and customized Douglas bag gas analysis system, as previously described (Russell et al., 2002). Peak aerobic capacity (V°O_2_peak) was calculated as the highest oxygen consumption recorded over a 30-s period. V°O_2_peak and MAP were used to calculate the PO for the individualized warm up on subsequent trial days, described below.

### Dietary control

CHO and caffeine intake were standardized the evening before and morning of a trial day and participants were also instructed to abstain from alcohol during the 24 h period prior to a trial day. Participants consumed an evening meal, snack and breakfast prepared by the team chef, providing a CHO content of 2, 1, and 2 g/kg BM, respectively. Participants were also provided with a post-exercise recovery drink, 20 min after the completion of the TT (1 g/kg BM CHO and 25 g protein). The composition and timing of all meals was repeated prior to trial two.

### Synthesis of ketone ester

The ketone ester synthesized, 1,3-butanediol acetoacetate diester, is a non-ionized sodium-free and pH-neutral precursor of AcAc (D'Agostino et al., 2012). The ketone ester was synthesized by transesterification of *t*-butylacetoacetate with *R,S*-1,3-butanediol (Savind, Seymour, IL). The resultant product consisted of a mixture of monoesters and diester, the ratio of which could be adjusted by varying the stoichiometry of reactants. After synthesis the crude product was distilled under reduced pressure to remove all solvents and starting materials and the resultant ketone ester was obtained and assessed for purity using gas chromatography-mass spectrometry (GC-MS).

### Trial day procedure

#### Participant preparation

On each of the trial days, participants reported to the laboratory in a rested and overnight (10 h) fasted state, with the timetable creating a ~30 min time between individuals that was repeated on the subsequent trial day. The trial day protocol commenced with the placement of an indwelling cannula (22G; Terumo, Tokyo, Japan) into a cephalic vein while lying in a supine position to allow for repeat blood sampling. A fingertip sample of capillary blood was collected concomitantly with each cannula sample throughout each trial for immediate measurement of blood ketones (ß-hydroxybutyrate; FreeStyleOptium Neo, Abbott Diabetes Care, Doncaster, Australia). Following a resting blood sample (*t* = 0 min), participants were provided their standardized CHO breakfast including 200 mg caffeine (NO-DOZ Awakeners, Key Pharmaceuticals Pty Ltd, Macquarie Park, Australia), to mimic typical race preparations. Participants were instructed to consume the breakfast within 10 min, with a second blood sample being collected at *t* = 30 min. At *t* = 70 min, participants provided a urine sample, were weighed and fitted with a heart rate (HR) monitor. At this time, they ingested the first dose (250 mg/kg BM) of KET or PLAC, followed immediately by 200 mL diet cola. At *t* = 90 min participants were seated on the Velotron ergometer, blood samples (4 mL) were collected and participants consumed their second dose in the same manner.

#### Warm up protocol

Following the second KET or PLAC drink, participants completed a standardized 20-min warm up on the cycle ergometer. The set-up of the bike was performed by team mechanics to replicate each rider's unique bicycle position and was fitted with a calibrated (Gardner et al., 2004) SRM cycling power meter (scientific version, 8 strain gauge, Schoberer Rad Meβtechnik; Jülich, Germany), set to sample at 1-s intervals. The warm up consisted of 3 × 5 min at 50% ventilatory threshold (VT), VT1, and VT1 plus 50% of the difference between VT1 and VT2 (156 ± 14, 312 ± 28, 355 ± 29 W, respectively), followed by 5 min self-paced cycling. Venous and capillary blood samples were collected every 5 min and expired gas was collected continuously during the first 15 min of the warm up. Immediately following the warm up participants provided a urine sample and ingested an energy gel containing 50 mg caffeine (27 g CHO, PowerBarPowerGel). During this time (5 min), participants were free to complete their own preparations during which pre-TT blood samples were collected, participants were provided with standard pre-race instructions and the zero offset of the SRM crank was set according to manufacturer's instructions.

#### Cycling time-trial (world championship road cycling time trial simulation)

The TT consisted of a simulation of the 2017 Bergen World Championship TT course, based on global positioning system (GPS) mapping data (road altitude and distance) collected by the Orica cycling team staff (M. Quod, unpublished observations). Cyclists completed the 31.17 km TT as fast as possible and during the TT the only feedback provided to the participant was the distance covered (km), cycling gear-ratio (12–27/48–54) and road gradient (%). Participants were only informed of their TT results following the completion of both trials. HR was collected every 5 km and ratings of perceived exertion (RPE) using the Borg 6–20 scale and capillary blood samples were collected at 15.74 km and immediately post TT. Participants ingested 250 mL of commercially available 6% CHO drink (Gatorade) at 15.74 km, as this distance corresponded to the point identified by the cyclists as the most appropriate opportunity to drink on the actual course. Samples of venous and capillary blood, and urine, were collected immediately following the TT and participants were weighed. At *t* = TT + 20 min, blood samples were collected and participants consumed a recovery drink (1 g/kg BM CHO) and continued to rest quietly for a further 40 min. Blood samples were collected at *t* = TT + 40 min and *t* = TT + 60 min, with a final urine sample being collected at *t* = TT + 60 min. Following the removal of the cannula, participants participated in a semi-structured interview with a single researcher using a series of standard questions to probe perceived effort, motivation and comfort rating during the TT. When symptoms (e.g., gut discomfort and problems) were identified, a standardized Likert scale was used to quantify them into mild, moderate, or severe levels. On completion of the second trial, participants were asked whether they could identify the trial in which they received the ketone ester, and the trial in which they had performed best. The interview technique was used to probe levels of interest in using a ketone ester supplement in real competition.

### Analytical procedures

Capillary blood samples were analyzed for concentration of ketones and lactate (Lactate Pro 2, Akray, Japan). Venous blood samples were collected into 4 mL SST vacutainers with immediate analysis of a small aliquot for blood glucose concentrations (Cobas Integra 400 plus, Roche Diagnostics, Switzerland). This venous sample was then centrifuged at 1,500 g for 10 min at 4°C, and aliquots of serum were stored at –80°C for later analysis. Samples were analyzed for FFA concentrations using a non-esterified-fatty acid (NEFA) assay kit (Wako Pure Chemical Industries, Ltd, Osaka, Japan), βeta-hydroxybutyrate concentrations using a β-hydroxybutyrate assay kit (Sigma-Aldrich, Ltd, Australia) and acetoacetate (AcAc) concentrations using an acetoacetate assay kit (Abcam, Cambridge, UK), as per the manufacturer's instructions. Urine samples were analyzed for urine ketones (namely AcAc) using ketone reagent strips (Keto-Diastix, Bayer).

### Data analysis

Statistical analysis was completed using SPSS (version 20 for Windows; SPSS, Chicago, IL). Paired t-tests were used to analyze average PO, cadence, HR and change in BM in the TT. Blood, urine, PO, HR, cadence, RPE, and respiratory data from the two trials were analyzed using a linear mixed model (treatment × time; *n* = 10) with the exception of respiratory data which includes (*n* = 9). When analyzing respiratory gases, an RER > 1.0 was not included in analysis as participants were not in steady state (*n* = 1, stage 3 for KET and PLAC). Statistical significance was set at *P* < 0.05 and data is presented as mean ± standard deviation (*SD*). TT performance was also analyzed for magnitude-based effect sizes between conditions using a custom spreadsheet (Hopkins, 2006). Data were log-transformed to account for non-uniformity and effect sizes with 90% confidence intervals [effect size (ES) ± 90% CI] were calculated and classified as either trivial (–0.2 to 0.2 ES) small (0.2–0.6, ES), moderate (0.6–1.2 ES), or large (1.2–2.0 ES). Where the 90% CI overlapped small positive (0.2) and negative (0.2) values, the effect was considered to be unclear.

## Results

### Participant experiences

Eleven cyclists commenced this study, but one participant experienced severe side effects from KET ingestion during and after the warm-up, including prolonged vomiting and dizziness and was unable to complete the TT. This participant withdrew from further participation in the study. However, data for this participant have been provided in the following analysis in comparison to those of the other riders to investigate a possible explanation for the occurrence of these side effects. All participants reported gastrointestinal discomfort associated with the intake of the ketone diester. Symptoms ranged from major (dry retching and nausea; *n* = 2), to moderate nausea (*n* = 5) or moderate reflux (*n* = 1), and minor discomfort (mild nausea; *n* = 2). No similar symptoms were reported with the PLAC trial. All participants correctly nominated the trial in which they received KET, identifying it via the gastrointestinal side-effects. However, only four of the cyclists correctly identified the trial in which they completed the TT in the fastest time, with one cyclist equivocal. Although each of the riders nominated their gut symptoms as a distraction or interference to performance, six participants identified an “unusual” centrally-derived feeling during the TT in the KET trial that they thought might be associated with better performance. When asked if they would use the current KET supplement in actual competition, prior to the unmasking of performance results, only one participant (who reported the least degree of discomfort during his KET trial) nominated being “possibly” interested. The remaining participants identified the need to remove the potential for illness and gut upset as well as to be sure of a robust performance effect before KET would be of value; “racing is hard enough without adding this complication.”

### Performance

**Figure 2:**
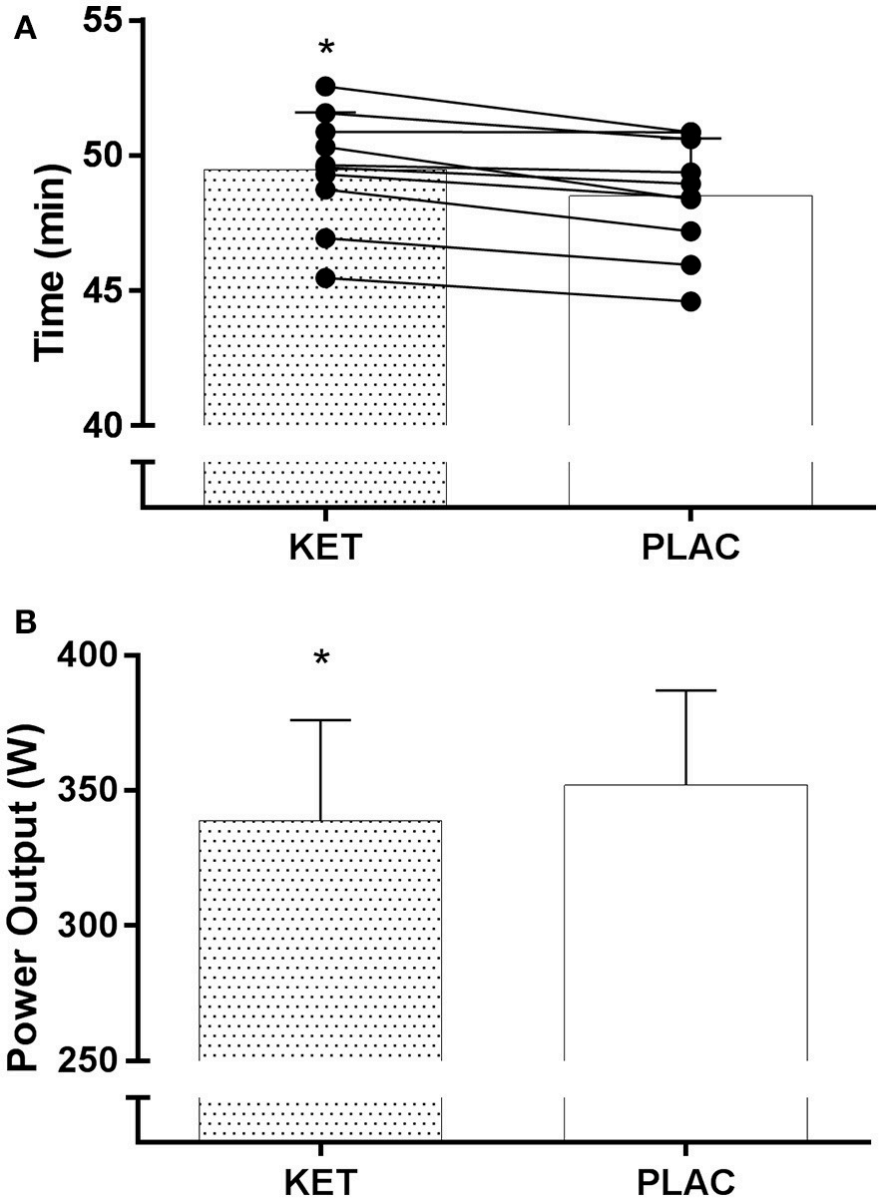
Mean and individual TT performance time **(A)** and mean power output **(B)** following exogenous KET or PLAC ingestion. Values are mean ± *SD*. ^*^KET different to PLAC.

**Figure 3:**
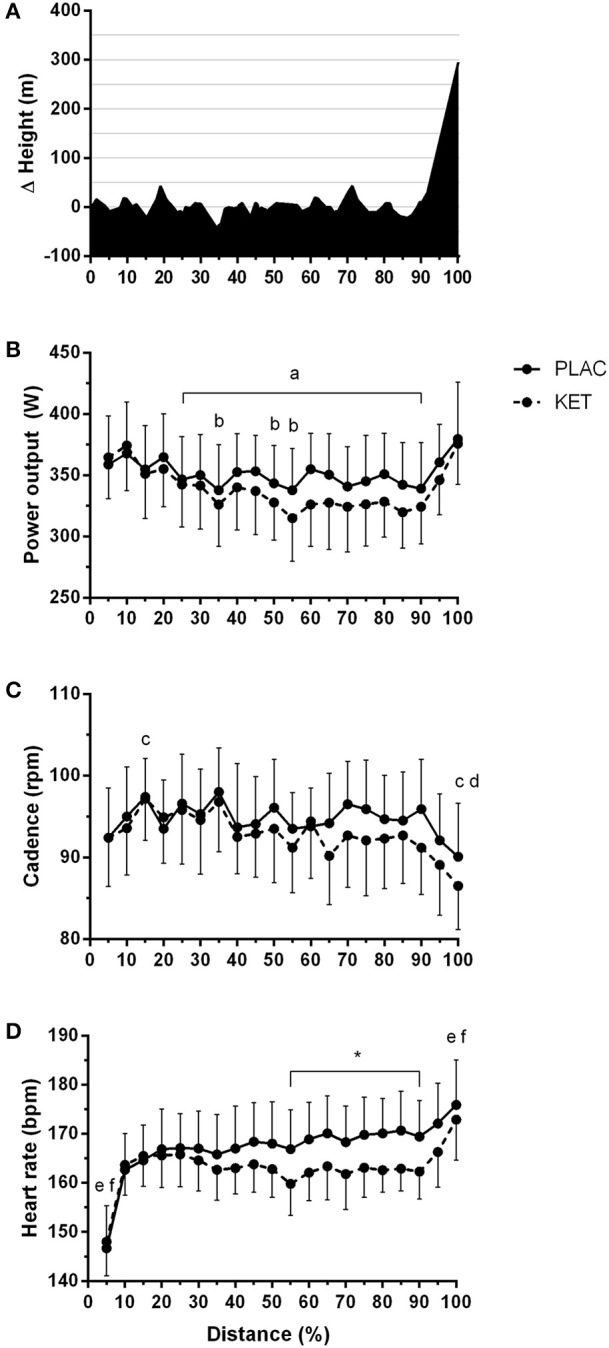
Course profile of World Championships TT course (Bergen, Norway) **(A)**, average power output **(B)**, cadence **(C)**, and heart rate **(D)** during TT as a percentage of total distance, following exogenous KET or PLAC ingestion. Values are mean ± *SD*. a different to 5% in KET; b different to 5% in PLAC; c different to 10% in KET; d different to 10% in KET; ^*^KET different to PLAC, e different to all other time points in KET, 50% different to f.

All cyclists completed the TT in a faster time in PLAC compared with KET, with the crossover allocation of treatments meaning that there was no order effect on performance. Figure 2 displays the results of the cycling TT including group mean and individual performances. There was an impairment to overall performance time with KET (2 ± 1%, 58.2 s; small ES –0.42 ± 0.1, *P* < 0.001). There was an impairment in cycling performance time in the first segment of the course (0–18 km; *P* < 0.001) and second segment with the climb included (18–32 km; *P* = 0.004) with KET ingestion compared to PLAC. Overall the KET condition was associated with a 3.7% reduction in average PO (KET 339 ± 37 W vs. PLAC 352 ± 35 W, *P* < 0.001, Figure 3B) and a lower cadence (KET 93 ± 6 rpm, PLAC 95 ± 6 rpm, *P* = 0.06, Figure 3C) compared to PLAC. There was an effect of time (*P* < 0.001) for power output and cadence during the TT, as displayed in Figure 3. A time × treatment interaction was reported for HR (*P* = 0.001) and average HR was significantly lower in the KET compared to PLAC condition (163 ± 7 vs. 167 ± 9 bpm, respectively, *P* < 0.01; Figure 3D). RPE increased in both the KET (16 ± 2 to 19 ± 2) and the PLAC (15 ± 2 to 19 ± 1) trials from mid- to post-TT (*P* < 0.001). There was no difference in RPE between trials despite the lower HR and PO in the KET condition.

### Serum metabolites

**Figure 4:**
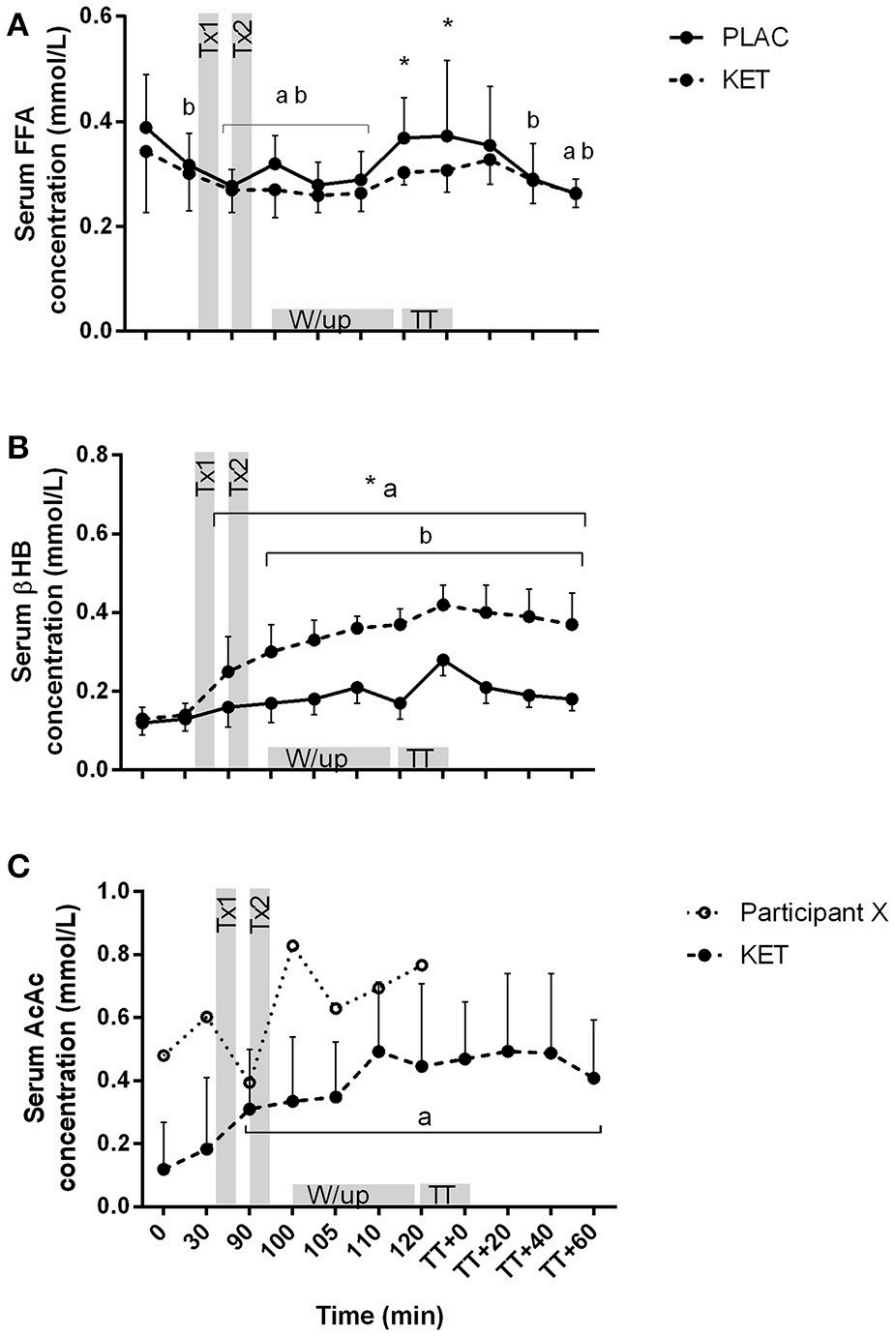
Serum FFA **(A)**, βHB **(B)**, and AcAc **(C)** concentrations following exogenous KET or PLAC ingestion whilst completing a cycling TT. Values are mean ± *SD*. Tx1 and Tx2 refer to dose one and two of KET or PLAC drink. ^*^KET different to PLAC at time point; a different to *t* = 0 min within KET; b different to *t* = 0 min within PLAC.

There was an effect of time (*P* < 0.001) and treatment (*P* = 0.021) for serum FFA concentrations (Figure 4A). FFA concentrations reduced from *t* = 0 during the 90 min following the CHO breakfast in both KET and PLAC trials (0.37 ± 0.10 mmol/L to 0.27 ± 0.04 mmol/L, *P* < 0.02). FFA concentrations were higher in PLAC vs. KET from pre- to post-TT (*P* < 0.05). A condition × time interaction was reported for serum βHB concentrations (*P* < 0.001; Figure 4B). There was an increase in βHB concentrations in the KET trial following dose one of KET ingestion (*t* = 90 min) and βHB remained significantly higher than PLAC trial until *t* = TT + 60 min. An increase in βHB concentrations from *t* = 0 was measured in the PLAC trial at the onset of the warm up (*t* = 100 min; Figure 4B) however βHB remained lower than in the KET trial. Serum AcAc concentration significantly increased from *t* = 0 min following dose one of KET ingestion (*P* = 0.001) and remained higher until *t* = TT + 60 min (Figure 4C).

### Capillary blood and urine metabolites

**Figure 5:**
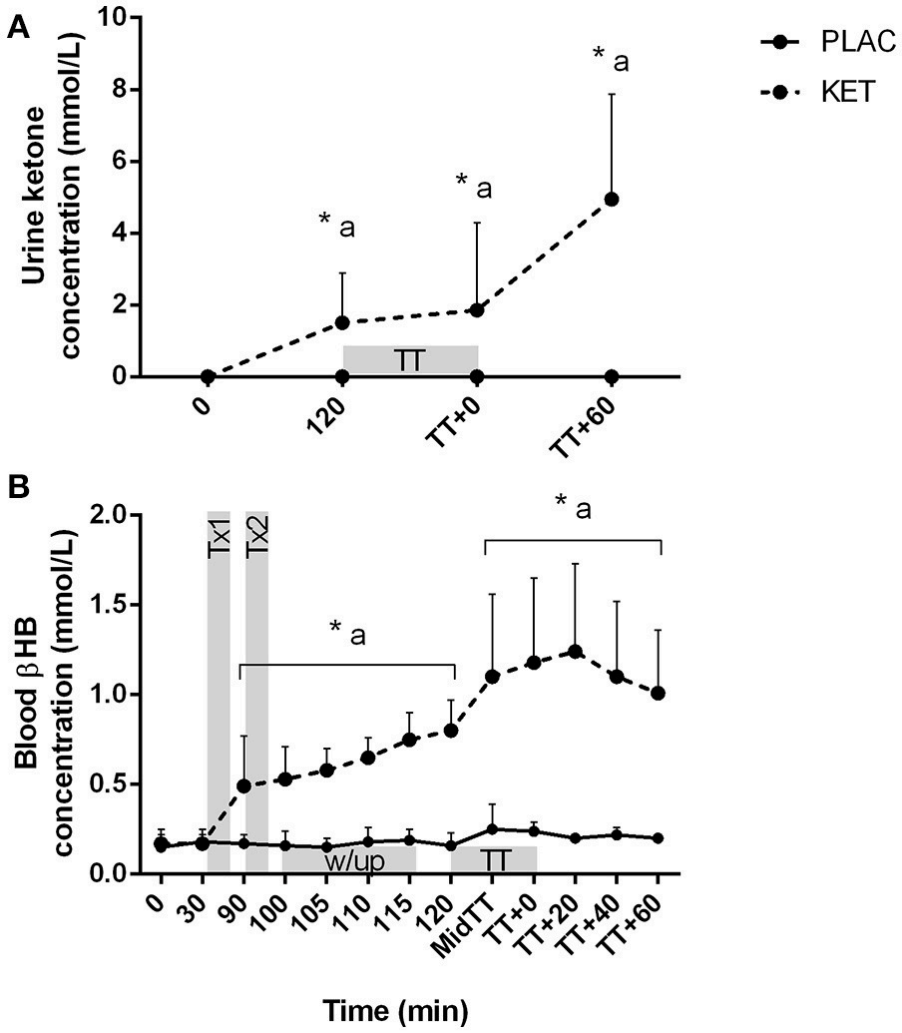
Urine ketone **(A)** and blood βHB **(B)** concentrations following exogenous KET or PLAC ingestion whilst completing a cycling TT. Values are mean ± *SD*. Tx1 and Tx2 refer to dose one and two of KET or PLAC drink, w/up refers to warm-up. ^*^KET different to PLC at time point; a different to *t* = 0 min within KET.

There was a condition × time interaction for both urine ketone and capillary blood βHB concentrations (*P* < 0.001; Figures 5A,B). No differences were observed at *t* = 0 between KET and PLAC for urine ketone concentration, but following KET ingestion, urine ketones were higher at pre-TT, post-TT, and at *t* = TT + 60 min for KET. Blood βHB concentrations increased following the first dose of KET, compared with PLAC ingestion (0.32 ± 0.16 mmol/L, *P* = 0.001). Blood βHB concentrations increased from pre-TT to post-TT in the KET trial (*P* < 0.001) and this increase was maintained until *t* = TT + 60 min (*P* = 0.03; Figure 5B).

**Figure 6:**
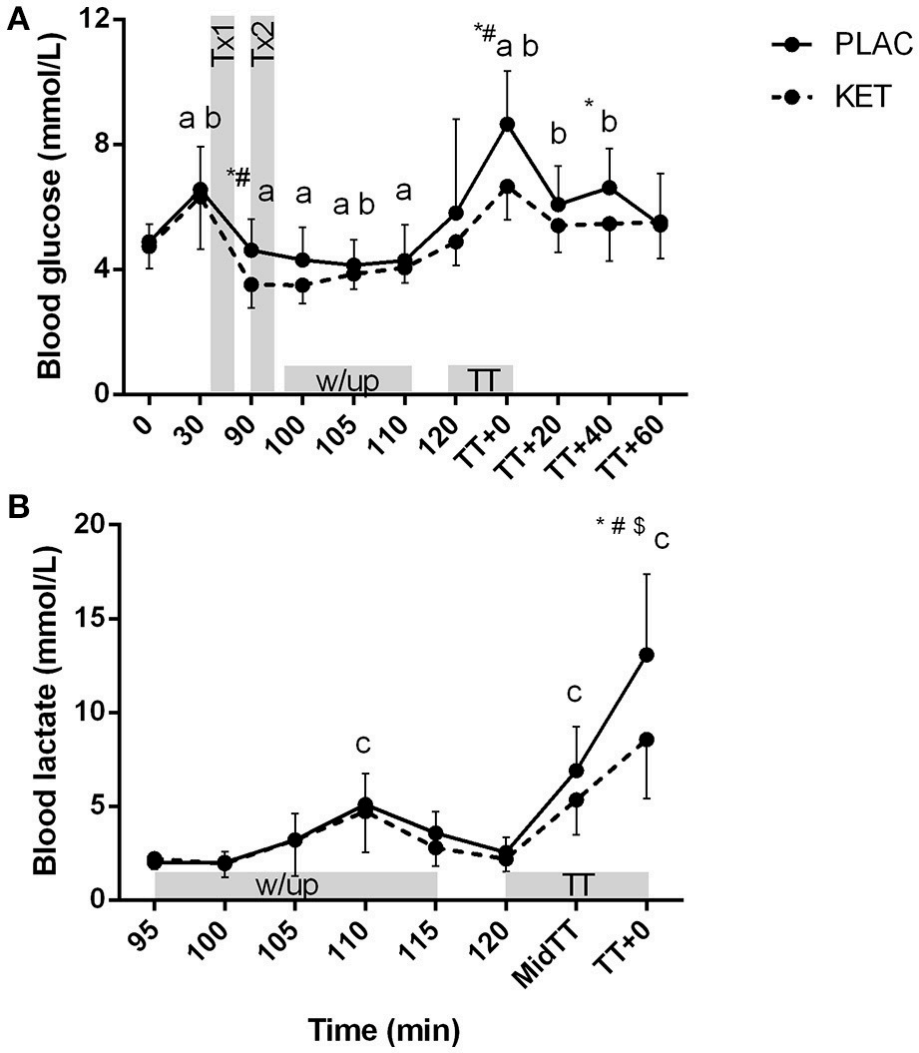
Blood glucose **(A)** and lactate **(B)** concentrations following exogenous KET or PLAC ingestion whilst completing a cycling TT. Values are mean ± *SD*. Tx1 and Tx2 refer to dose one and two of KET or PLAC drink. ^*^KET different to PLAC at time point; a different to *t* = 120 min within KET; b different to *t* = 120 min within PLAC; c different to 95 min within and PLAC; ^#^different to all other time points within PLAC; ^*$*^different to all other time points within KET.

There was a significant condition × time interaction for blood glucose (*P* = 0.036) and lactate concentrations (*P* < 0.001; Figures 6A,B). Blood glucose concentrations were lower in KET following the first dose of KET ingestion, pre-TT and *t* = TT + 40, compared to PLAC. Blood lactate concentrations increased from pre-warm up at the end of stage 3 (*t* = 110 min) in both the KET and PLAC trials but had returned to resting values pre- TT for both trials. Post-TT, blood lactate concentrations were significantly lower in the KET trial compared to the PLAC trial (8.6 ± 3.2 vs. 13.1 ± 4.3 mmol/L, *P* < 0.001, respectively).

### Respiratory parameters and BM

There was a main effect of time in the KET and PLAC trials for VO_2_, VCO_2_, and RER (*P* < 0.001), where an increase was observed throughout the incremental warm up from stage 1 to stage 3 (Table 1). There was a main effect of time for BM (*P* < 0.001) where a similar loss was measured in the KET (1.6 ± 0.7 kg) and PLAC (1.4 ± 0.4 kg) trials.

**Table 1:** Metabolic measures collected during 3 stage incremental warm up following exogenous KET or PLAC ingestion.

**Variable**	**Treatment**	**Stage 1**	**Stage 2**	**Stage 3**
VO_2_ (L/min)	KET PLAC	2.27 ± 0.27 2.33 ± 0.26	3.95 ± 0.37^*^ 4.01 ± 0.38^*^	4.42 ± 0.37 4.45 ± 0.42
VCO_2_ (L/min)	KET PLAC	1.94 ± 0.21 2.02 ± 0.24	3.66 ± 0.37^*^ 3.70 ± 0.28^*^	4.28 ± 0.38 4.31 ± 0.43
RER	KET PLAC	0.86 ± 0.04 0.87 ± 0.03	0.93 ± 0.03^*^ 0.93 ± 0.04^*^	0.96 ± 0.02 0.96 ± 0.03

* *Different to stage 1 and stage 3; Values are mean ± SD*.

## Discussion

This is the first study to report the effect of pre-exercise supplementation with a ketone diester on the performance of a cycling TT under conditions simulating real-life competition: laboratory simulation of a World Championship TT course in world-class male road cyclists who undertook nutritional strategies mimicking competition practices with respect to CHO and caffeine supplementation. Although, our protocol achieved hyperketonemia, as evidenced by increases in serum βHB and AcAc concentrations, there was an impairment of TT cycling performance in these elite cyclists. This outcome appears to be linked to the general observation of gut discomfort and intolerance among the study participants, with symptoms ranging from mild to severe. Our investigation adds important information to sports nutrition, by adding a real-world element.

The primary aim of our study was to address recent reports of enhanced sports performance associated with an acute increase in blood ketone concentrations following the intake of a ketone ester drink (Cox et al., 2016), by re-examining this concept in a more ecologically valid protocol. The investigation was undertaken as a collaborative project with a World Tour professional team of the International Cycling Union (UCI), offering an opportunity for them to make an evidence-based decision regarding the potential use of a highly discussed performance aid (Abraham, 2015). A number of features were included in the study design to optimize the reliability and validity of data, including opportunities to mimic the conditions under which the performance aid (ketone ester supplement) would be used. Team sports scientists were able to provide a simulation of the profile of the 2017 World Championships TT course (Bergen, Norway) on a cycle ergometer. Furthermore, the world-class cyclists from the team who participated in the study were personally motivated to receive individual and group results, and were highly experienced in TT cycling as well as familiarized to the specific laboratory-based course simulation. Race nutrition strategies (24 h prior, pre-race and during race) were standardized and made realistic by involving meal preparation by the team chef to suit both sports nutrition guidelines (Thomas et al., 2016) and the cultural practices of the riders. This included attention to achieve adequate CHO availability in pre-race meals (as opposed to the overnight fasted state involved in other studies such as Balasse, 1979; Fery and Balasse, 1986; Cox et al., 2016), as well as the real-life intake of caffeine and CHO supplements during the pre-race and within-race practices.

Our primary finding of a 2.0 ± 1% (58 s) longer time to completion in the TT following ketone ingestion supports our initial hypothesis that ketone ingestion would not enhance TT performance (Figure 2). Although, the gut disturbances were the likely cause of the performance impairment, we note that none of the cyclists achieved a faster time in the ketone trial, even when they reported very minor symptoms. Furthermore, the RPE in the ketone trial were similar to those on the placebo trial despite a lower power output and heart rate. This suggests that the gastrointestinal discomfort and/or some direct effect of ketones on the brain increased the perception of effort, and in accordance with the psychobiological model of pacing (Pageaux et al., 2014), our highly experienced cyclists reduced their work output to enable the TT to be completed without premature exhaustion. This finding warrants further investigation to confirm and explore the mechanisms. In the meantime, we note that the outcome of impaired performance with ketone ingestion is in contrast to previous studies (Clarke and Cox, 2015; Cox et al., 2016). Indeed, Clarke and Cox (2015) and Cox et al. (2016) reported 1–2% improvements in 30 min rowing performance and 30 min TT performance, respectively, following ingestion of a similar ketone dose to the current study (573 vs. 500 mg/kg BM, respectively) in combination with CHO, compared to CHO alone.

Due to the lack of a commercial supply, we were unable to obtain the ketone monoester supplement used in the study of Cox et al. (2016). However, we were able to source a diester that is currently being investigated as a potential treatment for seizures resulting from central nervous system oxygen toxicity (D'Agostino et al., 2013) and used in a similar dose to Cox et al. (2016). This diester contains a racemic mixture of βHB (i.e., contains both D- and L- enantiomers of the βHB) and has the ability to elevate both βHB and AcAc in a 1:1 ratio. The use of enzymatic analysis in the current study measures only the D- enantiomer which is the main circulating form of βHB and the most likely to have a direct effect on substrate metabolism and skeletal muscle responses (Yamada et al., 2010).

We provided the ketone drink in two doses, with the first bolus ingested 70 min prior to the TT. Based on previous research, our aim was to reach peak βHB concentrations at ~1 h following ingestion (i.e., prior to the TT; Pinckaers et al., 2017). We measured a modest increase in serum βHB concentrations in the ketone trial, reaching >0.3 mmol/L following the warm-up, but to our surprise serum βHB concentrations peaked immediately following the TT (>0.4 mmol/L; Figure 4B). The capillary whole blood samples analyzed for βHB concentrations measured values 2- to 3-fold greater than the serum samples (Figure 5B). This variation in D-βHB concentrations via enzymatic analysis (serum) and whole blood is consistent with previous literature reporting a –0.5 to 0.6 mmol/L higher concentration with handheld monitors (Pineda and Cardoso, 2015). This variation in measuring blood ketones in a controlled laboratory setting highlights the challenges athletes face in the field when aiming to reach and stay within the “optimal” range for a performance benefit (Egan and D'Agostino, 2016). We also measured a peak in serum AcAc concentrations following the warm-up, reaching ~0.5 mmol/L (Figure 4C). Therefore, when we consider total circulating ketones measured (i.e., βHB and AcAc) and the L- enantiomer that has not been measured, it is likely that athletes would be in the “optimal” range of 1–3 mmol/L for a proposed performance benefit (Egan and D'Agostino, 2016). Although “nutritional ketosis” was achieved, βHB concentrations reported in the current study are much lower than those reported previously (Cox et al., 2016). Cox et al. (2016) reported an increased in βHB concentrations to ~2 mmol/L within 20 min of ketone ester ingestion when co-ingested with CHO or ~4 mmol/L when ingested alone. This variation in serum βHB is likely explained by a range of factors including the different ketone esters used, the elite training status of cyclists in the current study and the different pre-ingestion nutritional strategies were the current study focused on appropriate race preparation practices.

Although we have not measured the same increase in circulating ketone concentrations as (Cox et al., 2016), ketones increased appropriately to alter metabolic responses compared to when a placebo was ingested. Blood glucose concentration was lowered in the ketone trial by ~1 mmol/L within 30 min following ingestion of the first ketone dose, and following the TT blood glucose was ~2 mmol/L lower than in the placebo trial. Additionally, we reported a 4.5 mmol/L (35%) reduction in blood lactate concentration following the TT in the ketone trial compared to the placebo trial. These findings of reduced blood glucose and blood lactate concentrations are consistent with the data of Cox et al. (2016) during 60 min of exercise at 75% Wmax and following a 30 min TT, respectively. We also measured lower circulating FFA during the TT following ketone ingestion compared to a placebo. Participants were cycling at 340–350 W during the TT and thus estimated contribution of FFA oxidation to total energy expenditure would likely be low as at this intensity, the muscle relies predominately on CHO-based fuels (Hawley and Leckey, 2015). However, the difference in FFA concentration between the ketone and placebo trials could be related to circulating ketone bodies having the ability to suppress lipolysis via inhibition of catecholamine's (Bjorntorp and Schersten, 1967).

As ketone bodies can be readily oxidized by skeletal muscle, expired gas was collected during the incremental warm-up. No differences in RER were measured between the ketone and placebo trials, although this could be related to the absolute exercise intensities attained by our elite subjects (155, 310, 355 W). Alternatively the high ketone concentrations in the urine suggest that the ketones are not being oxidized at the skeletal muscle. We have not estimated rates of substrate oxidation rates due to βHB and AcAc yielding respiratory exchange quotient values of 0.89 and 1.00, respectively and thus without appropriate correction factors for CO_2_ displacement and urine volume this would lead to an inaccurate representation of substrate utilization (Frayn, 1983; Pinckaers et al., 2017). We also reported a modest reduction in heart rate during the TT in the ketone trial compared to the placebo trial (5 bpm) which may be associated with a slightly reduced average power output and in the ketone trial.

Of the 10 participants who completed the trials, all reported gastrointestinal discomfort associated with the intake of the ketone ester including dry retching, mild to moderate nausea, reflux and minor discomfort. Furthermore, one participant was unable to start the TT due to prolonged vomiting and dizziness. This participant also experienced the highest concentrations of serum AcAc concentration when compared to the other 10 participants (Figure 4C, participant X), suggesting bioavailability of the ketone diester may impact individual responses following ketone ingestion. Although the side effects of ketone esters are not frequently discussed in the literature, Clarke et al. (2012) has provided evidence that participants have experienced a range of adverse effects including vomiting, nausea, diarrhea, and abdominal pain. These side effects have been associated with high dose of ketone ester and the consumption of the ester with a milk-based drink (Clarke and Cox, 2015). While it is possible that different dosing strategies, or the use of a different ketone ester product might eliminate or greatly reduce the gut problems seen in the current study, it is unclear whether a performance enhancement could be expected with exogenous ketone use in sporting events undertaken under the conditions employed in our study.

In conclusion, the results of the current study show that ingestion of a 1,3-butanediol acetoacetate diester under conditions of optimal race nutrition (i.e., CHO fed) results in increases in βHB and AcAc concentrations. The diester was associated with gut discomfort and intolerance among the cyclists with symptoms ranging from mild to severe. Despite optimal nutritional support (i.e., CHO breakfast, feeding during the TT and caffeine ingestion) for performance, ketone ingestion was associated with an increase in perception of effort, leading to an impairment of TT performance in elite professional cyclists.

## Author contributions

JL, MR, MQ, and JH: Conception and design, Collection and assembly of data, Data analysis and interpretation, Manuscript writing, Final approval of manuscript (required). LB: Conception and design, Financial support, Collection and assembly of data, Data analysis and interpretation, Manuscript writing, Final approval of manuscript (required).

### Conflict of interest statement

The authors declare that the research was conducted in the absence of any commercial or financial relationships that could be construed as a potential conflict of interest.
